# The role of indigenous knowledge and cultural dynamics in immunization uptake in Africa

**DOI:** 10.11604/pamj.2025.52.168.46893

**Published:** 2025-12-17

**Authors:** Frankline Sevidzem Wirsiy, Clovis Nchinjoh Sangwe, Arrey Denis Ebot Ako-, Nancy Tahmo, Eugene Vernyuy Yeika, Clinton Njakoi Kwemu, Jean-Claude Kindzeka Wirsiy, Roseline Dzekem Dine

**Affiliations:** 1 Africa Centres for Disease Control and Prevention, Addis Ababa, Ethiopia,; 2 Cameroon Baptist Convention Health Board, Yaounde, Cameroon,; 3 Department of Epidemiology, College of Public Health, University of Nebraska Medical Center, Nebraska, USA,; 4 The Pandemic Fund Secretariat, 1818 H Street NW, Washington DC, USA,; 5 Shoreland Inc. at 933 N. Mayfair Rd, Milwaukee, WI 53226, Wisconsin, USA,; 6 Gavi, the Vaccine Alliance, Geneva, Switzerland,; 7 Division of Public Health, Faculty of Health Sciences, University of the Free State, Bloemfontein, South Africa,; 8 Catholic Relief Services, Brazzaville, Republic of Congo,; 9 Division of Epidemiology, Dalla Lana School of Public Health, University of Toronto, Toronto, Ontario, Canada,; 10 Health Systems and Programme, Ministry of Public Health, Yaounde, Cameroon,; 11 Biomedical Sciences and Health Program, Faculty of Interdisciplinary at Saint John, University of New Brunswick, New Brunswick, Canada,; 12 Department of Social Sciences and Community Engagement, Rinda Ubuzima, Kigali, Rwanda,; 13 Department of Health Research Methods, Evidence, and Impact, McMaster University, Ontario, Canada

**Keywords:** Indigenous knowledge, cultural dynamics, immunization, uptake, Africa

## Abstract

Despite fifty years of development in immunization programs, vaccine uptake is still inconsistent throughout many parts of Africa. As global public health experts with a background in epidemiology and control of infectious diseases, including community engagement, we think the secret to closing the gap lies in the indigenous knowledge and cultural dynamics. These factors, which have their roots in conventional systems, affect vaccine acceptability by influencing how people view health and illness. This viewpoint emphasizes how indigenous customs, cultural values, and contemporary vaccination methods interact. Using cultural rites, the roles of traditional rulers, traditional healers, and community gatekeepers as examples, we suggest incorporating these dynamics into standard immunization frameworks. This culturally sensitive technique will promote equal vaccine uptake among Africa's diverse communities by developing trust and bolstering vaccination resilience. Cultural and indigenous practices are key to understanding barriers to immunization uptake, especially in rural and underserved communities. This school of thought addresses how leveraging indigenous knowledge can strengthen routine immunization programs and improve resilience against vaccine hesitancy rooted in cultural misconceptions. To effectively incorporate indigenous knowledge and cultural dynamics into immunization frameworks, we propose a three-pronged approach dubbed, the ´LET´ perspective i.e. L= leverage cultural events, E=engage traditional elders and healers, T=train community healthcare workers.

## Perspectives

We are reminded of the unrealized potential of indigenous knowledge systems and cultural dynamics in public health as we consider 50 years of immunization programs in Africa, largely as a result of the Essential Program on Immunization (EPI) [1]. Our field experiences in community engagement and mobilization have demonstrated that traditional beliefs and practices are not barriers to expanding immunization uptake across the continent, but rather advantages. Instead of using vaccine hesitancy as a chance to address deeply ingrained cultural and traditional systems, global public health initiatives all too frequently view it as a problem to be solved [2, 3]. Because communities may view vaccination programs as outside initiatives that ignore their traditional health practices, this strategy may lead to resistance. However, rather than imposing foreign ´medical techniques´, we should reframe immunization efforts as a continuation of community-based healing traditions by using indigenous knowledge systems. For example, traditional healers, elders, and religious officials have a big say in health decisions in many African countries [4]. These community leaders are frequently the first people that indigenes approach when they need medical advice. Without these community leaders, there is risk for vaccine hesitancy, and this was exemplified during the vaccinations against COVID-19. Furthermore, a commentary [5] highlighted six major determinants for vaccine uptake summarized by the acronym VAMRIS: V=vaccine hesitancy, A=attitudes of healthcare workers, M=misinformation, R=religion, I=immunization roll-out plans, and S=social influences. Successful vaccine uptake will depend on community engagement, targeted health promotion, myth dispelling, endorsements from local/community leaders, logistical planning, and incentives for health workers [6].

Working with these reliable individuals has changed vaccination campaigns, as we have seen in our work [7]. Acceptance rises when traditional leaders promote immunizations using terms and analogies that their people are accustomed to [8]. By using these reputable voices to spread the word about the advantages of vaccination, we can foster trust. This will greatly contribute to addressing false information declared by the World Health Organization (WHO) as an Infodemic [9]. Additionally, cultural events and rituals offer a way to publicize vaccines in a socially acceptable context [8]. Some cultures use initiation ceremonies, which include health-related customs, to mark the passage to adolescence and/or adulthood [10]. Vaccines might be seen as necessary life milestones rather than medical mandates by integrating immunization into such rituals. When immunization programs are in line with regional traditions, like combining child vaccination with naming ceremonies or community festivals, the outcomes are remarkable [11]. African traditions also use storytelling as a potent weapon. Many groups use folktales, proverbs, and oral histories to transmit knowledge. People respond strongly to health communication tactics that employ culturally appropriate storylines [12]. Sharing success stories of families whose children have received vaccinations to prevent sickness, for instance, can have a significantly greater impact than merely presenting numbers. In light of Africa's five decades of vaccination history via EPI, we envision a future in which cultural dynamics and indigenous knowledge play a key role in global public health initiatives. We must acknowledge traditional ideas as significant assets rather than as barriers. Co-creating public health plans with communities and ensuring that vaccination becomes a culturally accepted and embraced habit will be the key to making real progress in vaccine uptake [13], rather than imposing ‘external solutions’. The below schools of thought explore how integrating indigenous knowledge into immunization strategies-enhances more effective immunization programs:

**Indigenous knowledge as a lens for public health interventions:** Africans have historically used indigenous knowledge systems to manage illnesses and health [14]. Traditional healers provide treatments that are in line with cultural beliefs, making them the primary port of call for illnesses in many areas [15]. We have seen how communities place greater trust in local healers than in official medical institutions. These well-known individuals could be effective allies in debunking myths and promoting vaccination uptake if we include them in vaccine promotion.

**A double-edged sword: cultural beliefs:** cultural factors have the power to support or undermine vaccination campaigns [16]. Some groups, for example, consider vaccination to be alien, in opposition to their traditional beliefs about protection through natural exposure. We learned from this how crucial it is to match local values with health messages. We remember immunization campaigns in Cameroon in which communities opposed polio and the recent malaria vaccinations because of culturally motivated allegations. However, we witnessed a remarkable improvement by working with local leaders and presenting the malaria vaccine message in their cultural context during social mobilization activities such as door-to-door visits and announcements during religious services [17] which resulted in increased uptake of the vaccine.

**Catalysts for change: community gatekeepers and/or gate openers:** we frequently refer to community leaders as “gate openers” and “gatekeepers” in our work. They have the authority to shape group behavior since they are the keepers of tradition and trust. Involving community gatekeepers in vaccination efforts and community-engaged research [18] guarantees that the messages reach the individuals under their supervision. For instance, by facilitating community discussions, elders in some rural areas in Cameroon were instrumental in overcoming vaccine hesitancy and eventually increasing coverage. This is the case of the malaria vaccine introduction (MVI) in Cameroon, where despite strong governmental support, the initial launch of MVI, which was supposed to take place on December 12, 2024, was delayed due to the dissemination of false information claiming the malaria vaccine was harmful and ineffective after the first shipment was received [19]. Community leaders had to be highly involved to help avert the problem of vaccine hesitancy and misinformation, which was exacerbated by the COVID-19 pandemic, thus affecting MVI [20]. The Cameroon Ministry of Public Health and partners were able to increase community-based risk communication with health workers and community leaders instead of relying on the traditional mass media or other means. The involvement of community gatekeepers and/or gate openers contributed to the relatively successful launch of MVI in Cameroon. If, by the end of 2026, Cameroon aims to scale up the malaria vaccine to all its 205 districts [21], this will mean there is a need for a systemic inclusion of community gatekeepers and/or gate openers.

**Traditional roots, modern strategies:** while supply chains, logistics, and cutting-edge technology have been the emphasis of contemporary immunization programs [22], cultural issues must be incorporated for overall success. We learned this lesson during the COVID-19 pandemic, when vaccination efforts were hampered by false information that was frequently based on cultural narratives [23]. Modern science and conventional knowledge can be combined to build robust systems that ensure no one is left behind.

**A roadmap for integration; the LET framework as an organizing perspective:** to effectively incorporate indigenous knowledge and cultural dynamics into immunization frameworks, we propose a three-pronged approach dubbed, the L-E-T perspective i.e. L= leverage cultural events, E= engage traditional elders and healers, T= train community healthcare workers; which as an organizing perspective involves: i) L=leverage cultural events: this entails integrating immunization activities into traditional ceremonies, making vaccines an accepted part of cultural life [24]. ii) E=engage traditional elders and healers: developing partnerships and buy-ins with traditional health practitioners as well as community leaders and equipping them with accurate vaccine information to share with their communities [25]. iii) T=train community healthcare workers: training community healthcare workers as immunization staff to understand and respect cultural contexts, enabling them to communicate effectively with diverse populations [26].

## Conclusion

It is clear from looking back at 50 years of Immunization programs in Africa that cultural dynamics and indigenous knowledge systems are essential to vaccine acceptance and uptake rather than merely being incidental. Integrating traditional beliefs into immunization methods offers a chance to promote trust, community ownership, and long-term vaccine resilience rather than seeing them as obstacles. Working with traditional leaders, integrating vaccination into cultural rites, and using indigenous storytelling can all greatly increase vaccine uptake, as my experiences in community involvement have shown. The WHO has identified the problem of false information as an “infodemic,” which calls for culturally aware methods to combat. Using established social institutions, traditional healing methods, and cultural narratives can help make sure that immunization campaigns are well-received by the community. Traditional elders, healers, and gatekeepers in the community serve as “gate openers”-reliable individuals who, when actively involved, can promote vaccine uptake. The L-E-T organized perspective-Leverage Cultural Events, Engage Traditional Elders and Healers, and Train Community Healthcare Workers-serves as a strategic framework for embedding indigenous knowledge into immunization programs. By making vaccines an integral part of traditional ceremonies, empowering community leaders with accurate information, and training community healthcare workers in culturally respectful communication, we can create a public health framework that is not only scientifically sound but also socioculturally accepted. In order to overcome vaccine hesitancy and guarantee fair vaccine coverage, contemporary medical science and traditional knowledge must work in concert as Africa continues its immunization journey. Co-creating solutions with communities instead of enforcing outside interventions is the key to long-term success in immunization programs. By embracing cultural wisdom and indigenous practices, we can build resilient, inclusive, and sustainable immunization systems that leave no one behind.
